# Measuring change in activities of daily living in nursing home residents with moderate to severe cognitive impairment

**DOI:** 10.1186/1471-2318-6-7

**Published:** 2006-04-03

**Authors:** G Iain Carpenter, Charlotte L Hastie, John N Morris, Brant E Fries, Joel Ankri

**Affiliations:** 1 Centre for Health Services Studies, George Allen Wing, University of Kent, Canterbury, Kent, CT2 7NF, UK; 2 Research and Training Institute, Hebrew Rehabilitation Center for Aged, Boston, 1200 Centre Street, Massachusetts 02131, USA; 3 University of Michigan Institute of Gerontology, 300 North Ingalls Ann Arbor, MI 48109-2007, USA; 4 Centre de Gérontologie, Hôpital Ste. Périne, Paris, France

## Abstract

**Background:**

The objective of this study was to assess the responsiveness of the Minimum Data Set Activities of Daily Living (MDS-ADL) Scale to change over time by examining the change in physical function in adults with moderate to severe dementia with no comorbid illness who had been resident in a nursing home for over 90 days.

**Methods:**

Longitudinal data were collected on nursing home residents with moderate (n = 7001) or severe (n = 4616) dementia in one US state from the US national Minimum Data Set (MDS). Severity of dementia was determined by the MDS Cognitive Performance Scale (CPS). Physical function was assessed by summing the seven items (bed mobility, transfer, locomotion, dressing, eating, toilet use, personal hygiene) on the MDS activities of daily living (ADL) Long Form scale. Mean change over time of MDS-ADL scores were estimated at three and six months for residents with moderate (CPS score of 3) and severe (CPS score of 4 or 5) dementia.

**Results:**

Physical function in residents with moderate cognitive impairment deteriorated over six months by an average of 1.78 points on the MDS-ADL Long Form scale, while those with severe cognitive impairment declined by an average of 1.70 points. Approximately one quarter of residents in both groups showed some improvement in physical function over the six month period. Residents with moderate cognitive impairment experienced the greatest deterioration in early-loss and mid-loss ADL items (personal hygiene, dressing, toilet use) and residents with severe cognitive impairment showed the greatest deterioration in activities related to eating, a late loss ADL.

**Conclusion:**

The MDS-ADL Long Form scale detected clinically meaningful change in physical function in a large cohort of long-stay nursing home residents with moderate to severe dementia, supporting its use as a research tool in future studies.

## Background

Dementia, characterised by a progressive decline in cognitive function, affects up to 11 percent of those 65 and over, and 25 to 47 percent of those over the age of 85 [1]. Dementia adversely affects physical function, as measured by dependence in activities in daily living (ADL) [2-6]. Such decline causes a substantial burden for patients, their caregivers and society as a whole as patients become more dependent on caregiver and paid professional support, leading to increasing costs of care [7]. A loss of independence in activities of daily living is a key determinant of patient health-related quality of life [8] and a predictor of institutionalisation and mortality [9-11].

Dementia is widely prevalent in nursing homes residents [12,13], however few studies have assessed how physical function changes over time in nursing home residents with different levels of severity of cognitive impairment. The relationship between cognition and ADL has become an increasingly important area of dementia research as it can help to inform treatment decisions and it can help in the understanding of the value of new generations of pharmaceutical agents which aim to enhance cognitive function [14]. Previous studies that have assessed change over time have described change during the first 90 days of stay and therefore incorporated the confounding effects of change immediately after admission to a nursing home [15]. Other investigations included residents with comorbid illness, thus confounding the extent of the association between dependence in ADL and severity of dementia [16,17].

The Minimum Dataset Set (MDS) provides extensive data on residents of nursing homes. It was introduced in the United States in the early 1990s in response to the United States Congress in the Omnibus Budget Reconciliation Act of 1987 [18] and includes the mandatory requirement of data collection on cognition and ADL. The MDS is a unified, comprehensive needs assessment system used by professionals from multiple disciplines. It was developed through clinical reviews, tested in nursing homes and evaluated for validity and reliability [18]. A full assessment is performed on admission and annually thereafter, with a reduced quarterly assessment done every 90 days. Since implementation nationwide in the US, the MDS assessment tools have been introduced for care needs assessment and care planning in many countries around the world.

Although the primary aim for the development of the MDS is to improve the quality of care in nursing homes, it also provides a unique database for researchers. A number of studies have assessed the validity of specific MDS rating scales and commented on their usefulness as research tools [19,20]. MDS rating scales for physical function have been validated against other standardised instruments, however few studies have assessed how well these MDS ADL scales measure change over time [19].

This study aims to assess the responsiveness of the MDS-ADL scale to change over time by examining the change in physical function in adults with moderate and severe dementia with no comorbid illness who have been resident in a nursing home for over 90 days. We excluded residents with very severe dementia as they were likely to be already severely physically impaired, and those with very mild dementia as decline in ADL was less likely to be related directly to cognitive impairment.

## Methods

### Study design and population

This study was a longitudinal analysis of a representative sample of nursing home residents in one US state. MDS assessments for residents with a length of stay of greater than 90 days in the year 2002 were collected from a US National Resident Assessment Instrument database (n = 66,742) and follow-up data were extracted at three and six months [21]. We chose three month intervals between assessments to enable more precise examination of the rate and direction of change than is available in many longitudinal studies with assessment intervals of a year or more [22,23]. Residents with an expectation of being discharged prior to 90 days were excluded (n = 1998).

In order to minimise the effect of factors, other than cognitive impairment, in relation to change in ADL, residents were excluded if they had specific co-morbid diseases or conditions, or received specific treatments. These included history of learning disability, decreased auditory and visual acuity, problems with expressive speech, hypotension, seizure disorder, traumatic brain injury, manic depression, schizophrenia, HIV infection, septicaemia, weight fluctuation, inability to lay flat due to shortness of breath, dehydration, insufficient fluid consumption, internal bleeding, recurrent lung aspiration, end stage of disease, feeding by parenteral/IV or feeding tube, treatment including chemotherapy, radiation, tracheotomy care, transfusion, ventilator, for alcohol/drug problems, hospice care and respite care. Residents with concurrent or pre-existing limitations to mobility such as hip fracture, missing limb, bone fracture, cerebral palsy, stroke, hemiplegia/paresis, multiple sclerosis, paraplegia, Parkinson disease, or quadriplegia were also excluded. After applying these exclusions, the total number of residents available for study was 21,670. The most common reasons for exclusion were stroke (23%), presence of a learning disability (19%), impaired vision (10%) and hip fracture (5%). Nearly 40% of the sample had two or more of these exclusion criteria.

### Assessment of cognitive impairment

Cognitive impairment was measured using the MDS Cognitive Performance Scale (CPS), which classifies all residents into seven levels of cognitive performance ranging from a score of '0' (intact) to '6' (very severe impairment). These scores are derived from four MDS variables – two cognitive items (short-term memory and decision-making), one communication item (ability to make self understood) and one ADL item (eating). The CPS measure of cognitive impairment correlates highly with well accepted and frequently used research rating scale scores, including Folstein's Mini-Mental Status Examination (MMSE) [19,24-26]. This study examined the patterns of change in ADL for those residents with moderate to severe cognitive impairment therefore the CPS was used to identify residents in two groups: those with a CPS score of 3 (mean MMSE 15) and classified as moderate impairment (n = 7001) and those with a CPS score of 4 or 5 (mean MMSE 5–6) and classified as severe impairment (n = 4616). This method of classification has been used in other studies [16,17]. Residents with CPS scores of 0, 1, 2 (mild cognitive impairment) and 6 (very severe cognitive impairment) were excluded from the analysis.

### Assessment of activities of daily living

Change in ADL was measured using the MDS-ADL scale, which has also been validated against a number of standardised instruments for measuring physical function [26-28]. This study used the ADL Long Form scale of the MDS which is composed of seven ADL items. This scale is the most sensitive to change over time of the three principal summary MDS-ADL scales available [29]. The seven items are outlined in Table 1. Each ADL item has six possible categories of response representing observation by a trained clinical professional over an assessment period of seven days. Assessments based on observed rather than self-reported physical function were considered to be more reliable with high-risk groups such as residents with cognitive impairment, given that discrepancies can arise between the two methods of data capture [30]. The categories of response for the MDS-ADL self-performance scores range from 0 (total independence) to 4 (total dependence). The MDS Long Form ADL scale is a sum of the responses to all the seven individual ADL items and has a range from '0' to '28', with higher scores indicating a greater dependence [29].

**Table 1:** The seven items included in the MDS-ADL Long Form scale.

Item*	**Description**
Bed mobility	This includes how a resident moves and turns their body position while in bed.
Transfer	This includes how a resident moves between surfaces such as a bed and chair.
Locomotion	This includes how a resident moves between locations in their room and corridor outside their room.
Dressing,	This includes how a resident puts on, fastens, takes off all items of street clothing.
Eating	This relates to how a resident eats and drinks, including other means of intake of nourishments such as tube feeding.
Toilet use	This includes how a resident uses a toilet, commode, bedpan or urinal and transfer on and off the toilet.
Personal hygiene	This relates to how personal hygiene is maintained, including combing hair, brushing teeth, washing and drying face and hands but excluding baths and showers.

* Categories of response to each item include: 0 (total independence or no or little help with an activity), 1 (supervision provided 3 or more times during last 7 days), 2 (limited assistance by staff with the resident highly involved in activity), 3 (extensive assistance by staff with the resident performing part of the activity), 4 (total dependence – full staff participation in activity during the entire 7 days) and 8 (activity did not occur). For this scale, category 8 was combined category 4.

Baseline ADL scores were documented for each long-stay resident, using data from their earliest MDS-ADL assessment. Follow-up was assembled from assessments collected three and six months after this baseline assessment. In this way, the study includes long-stay residents with a range of nursing home lengths of stay in excess of 90 days.

### Analytic approach

We report here on the mean score calculated at baseline and at the three and six month follow-up, both for the overall ADL scale and for each of the seven individual ADL items. Mean change was calculated from baseline to three and six months for both cognitive impairment groups, and we report mean change, standard deviations (SD) and 95% confidence intervals (CI).

### Ethical review

The data used in this study were extracted from an aggregated anonymised database and submission to a research ethics committee was not required.

## Results

### Sample characteristics and loss to follow-up

The demographics and status at the three and six month follow-up are presented in Table 2. The moderate impairment group had a mean age (SD) of 85.6 (8.9) years and 80% were female. The proportion of those with moderate impairment no longer in residency at three months follow-up was 7% increasing to 18% by the six-month follow-up. The severe impairment group had similar demographic characteristics (mean age of 85.4 (8.7) years and 80% female). Of this group 8% were no longer in residency at three months, of whom a third had died. At six months 21% were no longer in residency, of whom a further 20% had died. Other reasons for loss to follow-up included transfer to another nursing home or acute hospital (10% at 3 months and a further 5% at 6 months) and discharge home or to another non hospital or nursing home setting, but reasons were not known for about half those who were not still resident at the three and six month follow-up.

**Table 2:** Demographics and status at three and six month follow-up for long-stay residents with moderate and severe cognitive impairment

	Moderate Impairment (CPS 3) N = 7001	Severe Impairment (CPS 4,5) N = 4616
**Age (%)**		
< 75	906 (8.7)	383 (8.3)
75–84	2051 (29.3)	1477 (32.0)
85–94	3501 (50.0)	2216 (48.0)
95+	840 (12.0)	540 (11.7)
**Gender (%)**		
Male	1429 (20.4)	926 (20.1)
Female	5572 (79.6)	3690 (79.9)
**Status at 3 months follow-up (%)**		
Still resident in NH	6518 (93.1)	4254 (92.2)
Discharged or transferred	65 (0.9)	40 (0.9)
Deceased	160 (2.3)	125 (2.7)
Lost to follow-up	258 (3.7)	201 (4.3)
**Status at 6 month follow-up (%)**		
Still resident in NH	5752 (82.2)	3642 (78.9)
3642 (78.9)	138 (2.0)	87 (1.9)
Deceased	360 (5.1)	320 (6.9)
Lost to follow-up	751 (10.7)	567 (12.3)

### Mean level of physical function

**Figure 1:**
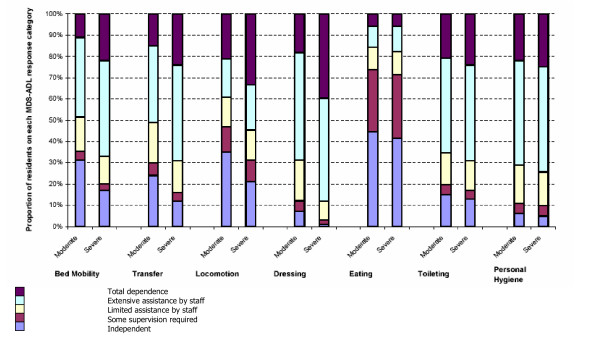
Baseline MDS-ADL long form item scores for residents with moderate and severe cognitive impairment.

In those with moderate cognitive impairment, mean ADL score at baseline was 14.77 (SD 7.34). The distribution of baseline scores for each ADL item is outlined in Figure 1. Dependence on staff was highest for functions relating to personal hygiene, dressing and toileting and lowest for eating. Mean ADL score increased to 15.63 (SD 7.40) at three months and 16.16 (SD 7.37) at six months (mean change at 6 months 1.8, std. dev. 4.4). For residents with severe cognitive impairment, the mean ADL score at baseline was 19.01 (SD 6.09). The distribution of ADL item scores for this group shows that dependence was highest for dressing, where 89% of residents required extensive or full assistance from staff, and lowest again for eating (Figure 1). The mean ADL score increased to 19.79 (6.16) at three months and 20.27 (6.07) at six months (mean change at 6 months 1.7, std. dev. 3.9).

### Change in physical function over time

Tables 3 and 4 show the change in ADL scores from baseline to three and six months for overall ADL score and for each of the seven individual ADL items. By the end of the six-month period, overall physical function of residents with moderate cognitive impairment deteriorated significantly by 1.78 points (95% CI; 1.67 to 1.91) and by 1.70 points (95% CI; 1.59 to 1.83) for residents with severe impairment. Each ADL item showed a significant worsening at both three and six months for both groups. However, although overall 56% and 57% of residents with moderate and severe impairment, respectively, showed a decline in physical function at six months, a quarter of residents in both groups showed an improvement. Fewer than 30% of all residents at three months and 20% at six months were relatively stable, i.e., not changing (positive nor negative) more than one point in the ADL scale.

**Table 3:** Mean change in physical function for each MDS-ADL Long Form item at 3 and 6 months for residents with moderate cognitive impairment (CPS = 3).

	Change baseline to 3 months (n = 6518)				Change baseline to 6 months (n = 5752)			
	Mean (SD)	95% CI	% decline	% improve	Mean (SD)	95% CI	% decline	% improve
ADL Long Form	1.02 (3.77)	0.92–1.08	45.8	27.0	1.78 (4.46)	1.67–1.91	55.6	24.8
Bed mobility	0.16 (0.93)	0.14–0.18	19.5	10.7	0.27 (1.06)	0.25–0.29	25.8	11.6
Transfer	0.17 (0.90)	0.15–0.19	20.1	10.7	0.28 (0.99)	0.27–0.31	26.6	10.3
Locomotion	0.22 (1.27)	0.20–0.24	20.8	11.5	0.33 (1.40)	0.30–0.38	27.4	12.7
Dressing	0.12 (0.73)	0.10–0.14	19.0	10.2	0.19 (0.82)	0.17–0.21	25.6	11.1
Eating	0.18 (1.00)	0.15–0.19	21.0	12.1	0.32 (1.13)	0.31–0.35	28.5	12.0
Toileting	0.12 (0.80)	0.10–0.14	18.3	10.8	0.24 (0.91)	0.21–0.25	26.4	11.2
Personal hygiene	0.11 (0.70)	0.09–0.13	18.9	10.2	0.21 (0.80)	0.19–0.23	26.8	10.5

**Table 4:** Mean change in physical function for each MDS-ADL Long Form item at 3 and 6 months for residents with severe cognitive impairment (CPS = 4 or 5).

	Change baseline to 3 months (n = 4254)				Change baseline to 6 months (n = 3642)			
	Mean (SD)	95% CI	% decline	% improve	Mean (SD)	95% CI	% decline	% improve
ADL Long Form	0.94 (3.30)	0.88–1.08	48.0	25.4	1.70 (3.87)	1.59–1.83	57.3	22.7
Bed mobility	0.17 (0.91)	0.14–0.18	20.7	10.4	0.27 (1.03)	0.23–0.31	27.5	11.1
Transfer	0.16 (0.87)	0.14–0.18	19.9	10.4	0.28 (0.97)	0.24–0.32	26.8	10.2
Locomotion	0.20 (1.21)	0.17–0.25	20.3	11.0	0.34 (1.35)	0.31–0.39	28.1	11.6
Dressing	0.07 (0.60)	0.05–0.09	15.6	9.8	0.14 (0.66)	0.12–0.16	21.9	10.1
Eating	0.27 (1.04)	0.25–0.29	29.1	13.0	0.46 (1.15)	0.41–0.49	38.2	11.9
Toileting	0.08 (0.67)	0.07–0.11	16.7	9.9	0.15 (0.72)	0.13–0.17	21.6	10.1
Personal hygiene	0.05 (0.54)	0.04–0.08	14.6	9.4	0.12 (0.63)	0.10–0.14	20.2	9.7

Residents in both cognitive groups showed a significant decline in all seven individual ADL items. The percentage of residents showing a decline ranged from 18% to 21% for the moderate cognitive impairment group and 14% to 29% for the severe impairment group at the three month assessment. This increased to ranges of 26% to 29% and 20% to 38% in the two cognitive groups, respectively, after six months. The percentage of residents who improved in each individual ADL item was smaller. Between 10% to 12% of those with moderate impairment and 9% and 13% in those with severe impairment improved at three months, and between 10% to 13% and 10% to 12% at six months (Tables 3 and 4).

There were also between-group differences in the decline rate for specific ADL's. A greater proportion of residents with moderate cognitive impairment experienced a decline in personal hygiene and dressing (early-loss ADLs items) and in toileting functions (a mid loss ADL item). At six months around 26% of residents with moderate impairment experienced a decline in these functions compared to around 21% with severe impairment. Although the proportion of residents showing a decline in the function of eating (a late-loss ADL) was high for both groups, it was highest for those with severe impairment (29% moderate vs. 38% severe).

## Discussion

This study was the first assessment of ADL change over time using the MDS-ADL Long Form scale in long-stay nursing home residents with moderate to severe cognitive impairment, with no comorbid illness. The MDS-ADL scale detected a mean decline in ADL for residents with moderate and severe impairment of 1.78 and 1.70 points respectively. A change of one point in the MDS-ADL scale denotes a clinically meaningful change. An increase of one point represents a precisely defined increased requirement for carer input confirmed as 'effective' during analysis and the extensive reviews and debriefings during the original development of the MDS-RAI [31]. Not only does this represent a change in the abilities of an individual, but also a change in the cost of their care. This study, which demonstrated a mean change of 1.7 points over six months based on a sample of 11,617 nursing home residents, shows that the MDS-ADL Long Form scale is sensitive to clinically relevant change over time in residents with moderate to severe cognitive impairment.

Furthermore, the study demonstrates that long-stay nursing home residents with moderate to severe cognitive impairment without comorbid illness have a high degree of physical impairment requiring assistance from staff in activities of daily living. Over time there is a general decline in ADL, indicating greater support is needed from nursing home staff. Comparison between the two dementia groups suggests a direct relationship between cognitive impairment and change in physical function over time; the more severe the cognitive impairment the more ADL items are likely to have been lost already, and the more likely they will continue to be lost in the ensuing months.

A higher proportion of residents with moderate cognitive impairment experienced a decline in the early and mid-loss ADLs (personal hygiene, dressing and toileting) compared to those more severely impaired. Residents with severe cognitive impairment showed the greatest decline in eating, a late-loss ADL. The main reason for the relatively small change in early and mid-loss ADLs (personal hygiene, dressing and toileting) for residents with severe impairment is likely due to these residents already having experienced substantial decline in these ADLs. This pattern of ADL follows the hierarchy of early, mid and late loss ADL originally outlined by Katz [32] and developed further in other studies since [18,33-35], and illustrated in the development of the MDS-ADL scales [29]. Early loss ADLs are in decline even before entering a nursing home and almost universally contribute to the reasons for admission [10,11,35].

Two other studies have assessed ADL change using MDS data. One study using the MDS-ADL 20-point scale in a sample of long-stay nursing home residents with CPS scores of 0 to 5 reported a mean change of 0.95 (SD 3.0) over one year [16]. Another study, using the MDS-ADL 24-point scale, demonstrated only a 0.15 (SD 7.0) change in residents with Alzheimer's' disease with at least one year of follow-up. It concluded that the MDS-ADL scale may be limited as an outcome measure as it would require in excess of 3000 patients to detect statistically significant changes [19]. However, this result was based on a very small sample size (n = 60). This present study, which demonstrated a clinically relevant mean change in MDS-ADL of 1.8 (SD 4.4) for the moderate and 1.7 (SD 3.9) for the severe group, suggests that statistical significance would require sample sizes of no more than 200 in each group, far fewer than the 3000 reported previously [19], further supporting its use as a research tool [36].

The main limitation of this study was that the analysis of ADL change over time does not include a number of residents who were lost to follow-up, either because they died or moved from the nursing home. However, this proportion was relatively small (19% at six months for both groups combined). Those residents who died may have shown a more marked decline in ADL compared to those who remained in the study. The study is also limited by the accuracy of the MDS dataset. However, for the cognitive function and ADL measures a high degree of reliability has been established [18].

This study supports existing evidence that despite the decline in ADLs, some residents showed an improvement in physical function – even long-stay residents with severe cognitive impairment. Investigation of factors associated with improvement and further research into interventions that reduce ADL dependency, such as rehabilitation therapy and the prescription of pharmaceuticals that alter the rate of decline in cognitive function, will have the added benefit of reducing the ever-increasing pressures on nursing home care staff and quality of life of cognitively impaired residents. As the MDS assessment instrument is widely used in routine clinical practice and has demonstrated utility for pharmacoepidemiology [37-40], further research on drug interventions for the cognitively impaired or dementia patients should examine the effects of change in physical function. It would build on evidence from recent studies that have reported the relationship between treatments and reduction in decline of activities of daily living [14] ensuring that the residents and their carers can gain maximum benefit from these new agents for a common, distressing and debilitating condition.

## Conclusion

Moderate to severe cognitive impairment was associated with decline in physical function, which confirms previous studies. However, further to this, we provide evidence of decline, and the rate of that decline, in a particularly vulnerable group in a relatively short period of time. This provides further supporting evidence for the use of the MDS-ADL scale as a research tool. The study also supports published research in the ordering of ADL loss into early, mid and late loss ADLs and its association with cognitive impairment. The more severe the cognitive impairment the more likely the subject is to have experienced decline in late loss ADLs. Most change in moderately impaired was in the early-loss and the mid-loss ADLs. These results could help direct the frequency and type of provision of care given to cognitively impaired residents of nursing homes and help inform future studies using MDS measures to assess the effect and cost benefits of interventions on the progression of dementia.

## Abbreviations

ADL Activity of Daily Living

MDS Minimum Data Set

CPS Cognitive Performance Scale

MMSE Mini-Mental Status Examination

## Competing interests

This study was supported by funding from Janssen-Cilag.

## Authors' contributions

GIC, CLH, JNM and BEF contributed to the concept, design, data analysis and interpretation and writing of the manuscript. JA contributed to the data analysis and interpretation and writing of the manuscript. All authors read and approved the final manuscript.

## Pre-publication history

The pre-publication history for this paper can be accessed here:


